# Identifying Novel Glioma-Associated Noncoding RNAs by Their Expression Profiles

**DOI:** 10.1155/2017/2312318

**Published:** 2017-09-12

**Authors:** Alenka Matjašič, Mojca Tajnik, Emanuela Boštjančič, Mara Popović, Boštjan Matos, Damjan Glavač

**Affiliations:** ^1^Department of Molecular Genetics, Institute of Pathology, Faculty of Medicine, University of Ljubljana, Ljubljana, Slovenia; ^2^Institute of Pathology, Faculty of Medicine, University of Ljubljana, Ljubljana, Slovenia; ^3^Department of Neurosurgery, University Medical Centre, Ljubljana, Slovenia

## Abstract

Long noncoding RNAs (lncRNAs) and microRNAs (miRNAs) play a significant role in cancer development as regulators of protein-coding genes. Their dysregulation was in some extent already associated with glioma, the most aggressive primary brain tumours in adults. The correct diagnosis and treatment selection due to high tumour heterogeneity might be difficult and inadequate, resulting in poor prognosis. Studies of expression patterns of noncoding RNAs (ncRNAs) could provide useful insight in glioma molecular development. We used the qPCR approach to screen and investigate the expression of lncRNAs that were previously deregulated in other cancer types. The study showed altered expression levels for numerous lncRNAs across histologically different glioma samples. Validation of few lncRNAs showed association of expression levels with histological subtype and/or malignancy grade. We also observed deregulated and subtype-distinctive expression for four lncRNA-associated miRNAs. Expression of few lncRNAs and miRNA was also associated with patients' survival, showing potential prognostic value. Several ncRNAs, some already related to glioma and some, to the best of our knowledge, investigated for the first time, might be of greater importance in glioma molecular development and progression. Finding the subtype-specific lncRNA and/or miRNA expression patterns may contribute additional information for a more objective classification.

## 1. Introduction

Long noncoding RNAs (lncRNAs) are a group of noncoding RNAs (ncRNAs), classified by the length longer than 200 bp ranging up to 100 kb, and do not appear to have coding potential [1, 2]. Based on their loci of origin, lncRNAs can be classified as intergenic/intronic, sense/antisense, bidirectional, or overlapping with protein-coding genes or other ncRNAs [3, 4]. Their lack of coding potential does not mean lack of function; their role in cancer development and progression was already confirmed, although mechanisms of their function are still not fully understood [5] and have yet to be investigated in detail. With progression in expression profiling, tens of functional examples have arisen that associate lncRNAs with diverse range of cellular processes [2, 3]. Mechanistically, various lncRNAs act as the cotranscriptional regulators of protein-coding genes [6] and are involved in various biological and developmental pathways, including the acquisition and maintenance of cell characteristics [7]. Certain lncRNAs were found to be closely associated with initiation, proliferation, invasion, and recurrence of glioma and may therefore be exploited for the purposes of subclassification, diagnosis, and prognosis [8]. Genetic and epigenetic changes that permit the survival of abnormal cells and formation of tumour mass often include genes that control critical biological processes and pathways, such as cell proliferation, adhesion, migration, and differentiation [9]. In tumour, deregulated cell proliferation is a critical event required for tumour to grow and invade, that is, for tumour progression [10]. Regulatory lncRNAs can intercede both tumour suppressor and oncogenic effects [3, 5, 11], and their aberrant expression emphasizes their role in variety of diseases [5, 12]. Altered expression of certain cancer type-specific lncRNAs can reflect disease progression [13] and might serve as potential biomarkers [11, 12]. Another interesting and also the most characterized group of ncRNAs are microRNAs (miRNAs), small, ~22-nucleotide long ncRNAs, which negatively regulate the expression of mRNA by binding on the untranslated mRNA and preventing their translation [14, 15]. In addition to lncRNAs, miRNAs can also function as tumour-suppressors or oncogenes. They can be expressed as single genes, gene clusters, or from introns of protein-coding as well as nonprotein-coding genes such as lncRNAs [16].

lncRNAs are widely expressed in mammalian nervous system [7], and several were identified to be specifically linked with neuro-oncological disorders [4, 17], including tumours of central nervous system (CNS) [7]. Glioma are the most common form of primary brain tumours in adults, and despite the progress in therapy, the prognosis of patients, especially those with glioblastoma (GBM), remains ruthless [18]. The lack of effective drugs and lack of inefficient treatment are probably consequences of glioma's heterogeneous and invasive nature. Regarding the latter, the exact pathways of glioma biogenesis and progression are thus still in question [19]. Numerous studies underline the association of changed expression profiles of specific lncRNAs with tumour histological grade [1, 3]. Aberrant lncRNA expression affects the expression of the target gene, which in turn leads to cell dysfunction and disease progression [2, 20], and may have a direct role in driving the disease state [3]. Indeed, altered expression of lncRNAs and miRNAs has been correlated with development of various cancer types and they could also help elucidate the mechanisms underlying glioma malignant transformation [12, 13]. In addition, as some glioma subtypes are known to have distinct molecular features [21], expression analyses of coding and noncoding genes can also provide additional information to further distinguish them (classification biomarkers) [13]. Only a few years ago, the golden standard for glioma classification has based mainly on their morphological features, including cells of origin, degree of differentiation, and grade of anaplasia [21]. However, histological diagnoses of glioma tend to be very difficult since many cases are morphologically alike, partly due to the lack of specific (particularly molecular) distinguishable markers [13]. The number of protein-coding genes only differs little between organisms, and as seen, they are not sufficient to properly explain the human gene expression complexity. It is suggested that the expression complexity could be explained right with these noncoding but transcriptionally active genes [22]. Certain lncRNAs, such as *H19*, *MALAT*, and *linc-POU3F3*, have been shown to be involved in cancer progression, and also glioma of different malignancy grades show different patterns of lncRNA expression [22–24]. Also, expression of miRNAs shows biomarker potential, used as diagnostic support or for prognostic and therapeutic application [25, 26].

The purpose of this study was to examine the lncRNA expression profile of 90 lncRNAs, previously reported to be involved in various types of cancer (Human LncProfiler™ qPCR Array Kit (System Biosciences, CA, USA)), in glioma samples and to characterize potential glioma-related lncRNAs. Expression of several selected, differentially expressed lncRNAs was validated on a bigger number of biological replicates. We searched which of validated lncRNAs might encode, be coexpressed, or coregulated with miRNAs. Those miRNAs were also analysed for potential correlation with their host or coregulated lncRNAs. At last, we evaluated the prognostic value of aberrant ncRNA's expression.

## 2. Materials and Methods

### 2.1. Tissue Samples and Clinicopathological Data

The study was approved by the National Medical Ethics Committee of the Republic of Slovenia (115/5/14). Sixty-four tumours that were surgically removed from the patients between 2008 and 2012 were chosen for the study. We randomly chose 17 glioma tissue samples for initial expression profiling from our previous glioma data set, with respect to appropriate RNA yield and integrity values. Expression validation set further included 60 glioma tissue samples, of which there were also 13 samples from the initial profiling set.

Tumours were stabilized in RNA*later* (Applied Biosystems, USA) immediately after surgical biopsy and incubated at 4°C for 7 days. After incubation, samples were stored at −20°C until RNA extraction, as recommended by the manufacturer. All tumours were evaluated by a neuropathologist in order to assess glioma subtype and tumour grade (see Table 1). The tumour biopsies used in the study belonged to 36 female and 28 male patients (median age at diagnosis: 51.8 years ± SD 16.2). As the expression study was conducted in the year 2012, sample classification is based primarily on a neuropathologist's evaluation of morphological features and previous diagnosis if existed and only in some cases based on the status of p53 and 1p/19q codeletion. Samples of diffuse and anaplastic astrocytoma were united in one subgroup, that is, grade II + III astrocytoma, for simpler comparison with other subtypes. For reference RNA, we used the FirstChoice Human Brain Reference Total RNA (cat. number 6050, Ambion, Invitrogen, USA) (further referred to as reference RNA), obtained from the brain tissue of 23 individuals without any signs of neurodegeneration.

### 2.2. RNA Extraction from Fresh Tissue

RNA from all the samples used in primary profiling set and validation set was extracted following the same isolation protocol. Total RNA from up to 30 mg of each tissue was isolated using TRIzol reagent (Invitrogen, USA), followed by purification using miRNeasy Mini Kit (Qiagen, Germany), according to the manufacturer's instructions. The yield was measured spectrophotometrically using NanoDrop-1000 (Thermo Scientific, USA), and the quality was evaluated on Bioanalyzer 2100 (Agilent Technologies, USA). After the RNA extraction and quality assessment, the samples with appropriate RNA yield and integrity values (concentration higher than 100 ng/*μ*L and RIN > 5.5) were considered for expression profiling and further validation analyses.

### 2.3. LncRNA Expression Profiling Using Human LncProfiler qPCR Array Kit

We performed a primary expression profiling of 90 lncRNAs that have already been associated with different types of cancer (Human LncProfiler qPCR Array Kit (System Biosciences, CA, USA)). lncRNA profiling of these 90 lncRNAs was performed using qPCR amplification of cDNA from an initial set of 17 glioma samples of different histopathological characteristics and normal brain reference RNA (control).

First strand of cDNA was synthesized using Human LncProfiler qPCR Array Kit with oligo-dT and random hexamers, according to the manufacturer's instructions. All qPCR reactions were carried out in triplicates and included a negative control. Quantification was performed using the ABI Prism 7900 sequence detection system (Applied Biosystems, USA). Cycling conditions were as follows: 2 minutes at 50°C, 10 minutes at 95°C, and 40 cycles of 15 seconds at 95°C, and one minute at 60°C. The signal was collected at the endpoint of each cycle. A cohort of five reference genes was simultaneously analysed for the normalization process (*18S rRNA*, *RNU43*, *GAPDH*, *LAMIN A/C*, and *U6 snRNA*), and the optimum reference genes were identified using NormFinder algorithm [27]. Expression values of lncRNAs in glioma were compared to values of normal brain reference RNA, and results were statistically analysed.

### 2.4. Validation of Selected lncRNAs

For more reliable results, results need to be validated on a larger number of biological replicates. Upon statistical analysis, we selected 7 lncRNAs (*EGO-A*, *7SL*, *RNCR3*, *MEG3*, *HOTAIR*, *ZFAS1*, and *JPX*) for validation and to further investigate their differential expression on a cohort of 60 glioma samples and normal brain reference RNA (control). For the subsequent analyses of lncRNA expression, cDNA was generated with Protoscript M-MuLV Taq RT-PCR kit using random primers (New England Biolabs, UK) according to the manufacturer's instructions, except were otherwise indicated. Reverse transcription reactions were prepared in 10 *μ*l master mix with 500 ng of total RNA. Expression analyses were validated using the TaqMan or SybrGreen-based qPCR technology, dependent on probes availability. All qPCR reactions were carried out on Rotor-Gene Q (Qiagen, Germany), and each sample was analysed in duplicate. Thermal conditions were applied as follows: initial denaturation at 95°C for 15 minutes and 40 cycles of denaturation at 95°C for 15 seconds and annealing at 60°C for 1 minute, and following amplification melting curves were acquired. The signal was collected at the endpoint of each cycle.

#### 2.4.1. TaqMan-Based Technology

qPCR reactions for *MEG3*, *RNCR3*, *JPX*, and *ZFAS1* were prepared with lncRNA-specific TaqMan assay (*MEG3* Assay ID Hs01098508_mi; *RNCR3* Assay ID Hs01039195_gi; *JPX* Assay ID Hs03681129_m1, *ZFAS1* Assay ID Hs03300756_m1) (all Applied Biosystems, USA) and FastStart Essential DNA Probes Master Mix (Roche, Germany) or TaqMan Gene Expression Master Mix (Applied Biosystems, USA) in 10 *μ*l reaction volume. Reference genes *GAPDH* and *18S rRNA* were analysed in both conditions.

#### 2.4.2. SybrGreen-Based Technology

qPCR reactions for *EGO-A*, *7SL*, and *HOTAIR* were prepared with designed, lncRNA-specific primers (*EGO-A:* F – CTTCTCCTCCAGGCCATACC, R – CCATTGTGTAGCCCCG; *7SL:* F – CTGTAGTCCCAGCTACTCG, R – CCCGGGAGGTCACCATATT; and *HOTAIR:* F – CAGTGGGGAACTCTGACTCG, R – GTGCCTGGTGCTCTCTTACC) and Power Sybr®Green PCR Master Mix (Applied Biosystems, USA) in 10 *μ*l reaction volume. *GAPDH* and *U6 snRNA* were used as reference genes.

### 2.5. Quantitative Real-Time PCR of miRNAs

miRNAs were analysed using either the miScript system (SybrGreen, Qiagen, Germany) or TaqMan-based technology (Applied Biosystems, USA) according to their corresponding lncRNAs. All the reagents were from Qiagen or Applied Biosystems, respectively, except where otherwise indicated. qPCR was carried out using the Rotor-Gene Q (Qiagen, Germany).

#### 2.5.1. miScript System (Qiagen, Germany)

All of the steps were performed according to manufacturer's protocol, except where otherwise indicated. miScript reverse transcription kit was used for RT in a 10 *μ*l reaction master mix with 100 ng of total RNA. All of the qPCR reactions were performed in duplicate, and following amplification melting curves were acquired. MicroRNAs *miR-196a* and *miR-125b* were tested relatively to *RNU6B* and *SCARNA17* (*RNU6B*, *SCARNA17*, *SNORA73A*, *and RNU1B* were tested as reference genes). The signal was collected at the endpoint of every cycle.

#### 2.5.2. TaqMan-Based Technology

All of the steps were performed according to manufacturer's protocol, except where otherwise indicated. *RNU6B*, *RNU48*, *RNU58A*, and *HY3* were tested as references genes. MicroRNAs *miR-124a* and *miR-770* were tested relatively to *RNU6B* and *RNU58A.* Briefly, the 10 *μ*l RT reaction master mix was performed with 10 ng of total RNA sample. The cDNA was diluted 30-fold, and 4.5 *μ*l was used for each 10 *μ*l qPCR reaction. The qPCR reactions were performed in duplicates, and the signal was collected at the endpoint of every cycle.

### 2.6. Computational and Statistical Analysis

Relative quantification of lncRNA and miRNA levels of the target gene was calculated using the ∆∆C_T_ and represents the difference between ∆C_T_ tumour RNA and ∆C_T_ reference RNA, normalized to expression of endogenous controls [28]. If ∆∆C_T_ was significantly (2*σ*) higher or lower than zero, the expression was considered to be significantly different. The calculation method was used for computational analyses for both lncRNA profiling and validation of differentially expressed lncRNA and miRNA.

Calculated ∆∆C_T_ values and the R programming language were used for unsupervised hierarchical clustering analysis, with Pearson correlation for distance metric. All statistical tests were performed using the IBM SPSS Statistics 20. software (IBM Corporation, New York, USA). Not all cases had all data available. Factor analysis was performed using the principal component extraction method and the direct oblimin as the rotation method. We used the Mann–Whitney 2-independent samples test to determine which of the 90 lncRNAs are differentially expressed, and results are graphically presented as log values on the heat map. For determining significant differences in expression (using ∆∆C_T_) of lncRNAs and miRNAs between glioma subtypes, we used the Kruskal-Wallis *k*-independent test (multiple comparisons). Mann–Whitney 2-independent samples test was performed to cross test expression differences between two subtypes. The expression differences were considered statistically significant when the differences in tested groups reached or were below *p* ≤ 0.05. The relative quantification of lncRNA and miRNA levels is graphically presented as average fold change (2^-∆∆CT^). The Pearson's correlation coefficient was used to define expression relationships and Kaplan–Meier estimate for constructing survival curves (analysed by log-rank test). To assess the relative risk for each factor, we performed the univariate and multivariate Cox regression analyses using R language. We used the ∆∆C_T_ values, and two-sided *p* value < 0.05 was regarded as significant.

## 3. Results

### 3.1. Profiling Shows Numerous lncRNAs with Altered Expression

**Figure 1:**
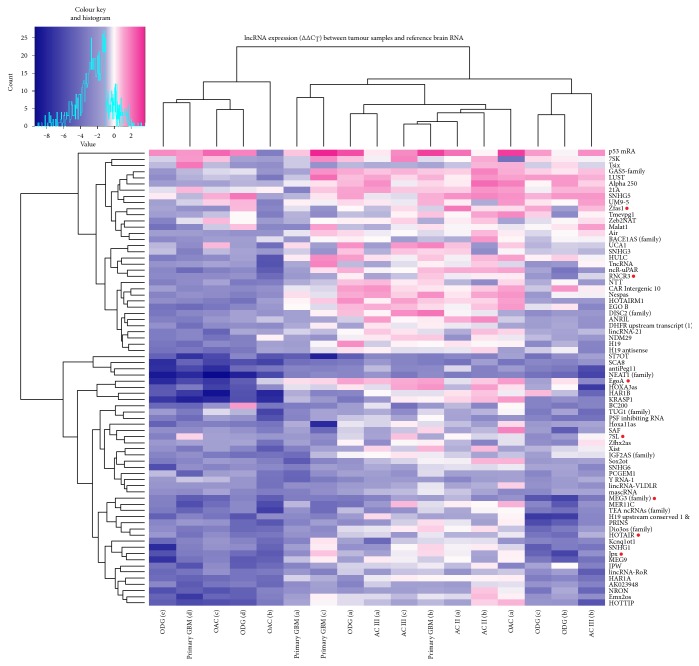
Heat map of lncRNA profiling analyses of glioma samples. The figure represents ∆∆C_T_ values of differentially expressed lncRNAs in glioma samples compared to human brain reference RNA. Data are presented on a colour scale where shades of blue represent decreased expression and pink as increased expression, with ∆∆C_T_ cut-off values set at −1 and 1. On the top of the figure is presented unsupervised Pearson's hierarchical clustering of samples. AC: astrocytoma of WHO grade II or III; OAC: oligoastrocytoma; ODG: oligodendroglioma. Genes denoted by red dot were selected for further qPCR validation and analysis.

**Figure 2:**
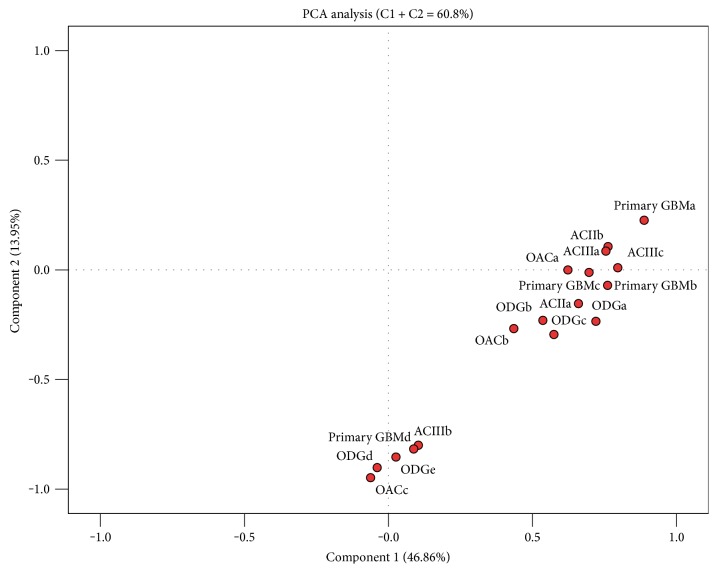
Principal component analysis of individual samples, generated with expression of 74 differentially expressed lncRNAs. AC: astrocytoma of grade II or III; GBMs: glioblastoma; ODG: oligodendroglioma; OAC: oligoastrocytoma. Letters at the end mean a consecutive sample.

lncRNA profiling revealed changes in expression levels for numerous lncRNA (74/90 lncRNAs analysed) in the primary set of 17 glioma samples compared to normal brain reference RNA (pooled RNA samples from 23 donors, commercially available). Unsupervised hierarchical clustering (Figure 1) upon similarity of gene expression profiles between tumour samples showed roughly three molecular groups; however, gene expression-based groups did not coincide with samples' histological subtypes, suggesting the heterogeneous lncRNA expression background of morphologically similar tumours. Factor analysis, with the principal component extraction method based on 74 gene expression profiles, showed two principle components, which together account for 60.8% of variance in gene expression (Figure 2). The component 1, accountable for 46.8% of variance, separates samples in two main groups, which remain more or less the same with component 2 (accountable for 13.95% of variance). Sample grouping again showing differences in lncRNA expression profile vary between glioma subtype and to some extent coincided with unsupervised hierarchical clustering (comparing Figures 1 and 2).

### 3.2. Expression Validation of Seven lncRNAs

**Figure 3:**
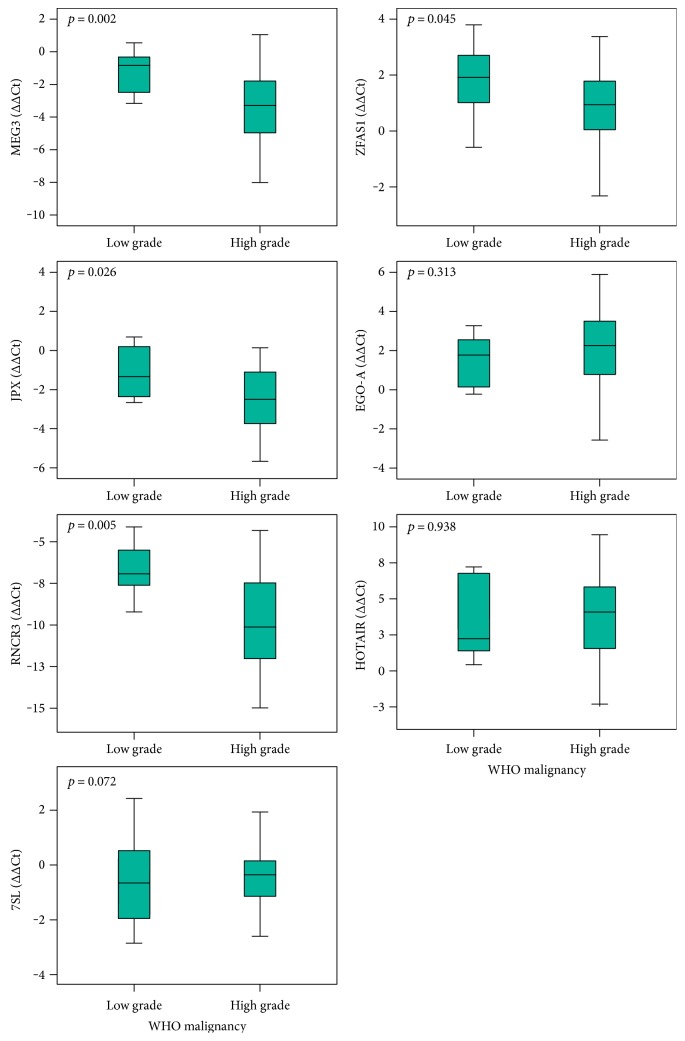
qPCR results of lncRNA gene expression regarding the WHO malignancy grade. Expression analyses of 7 lncRNAs, previously determined as differentially expressed, between low and high malignancy grade. Data are presented as ∆∆C_T_ box plots ± standard error.

Among differentially expressed lncRNAs, we further validated seven lncRNAs (*7SL*, *EGO-A*, *HOTAIR*, *JPX*, *MEG3*, *RNCR3*, and *ZFAS1*) on a bigger cohort of glioma samples of different WHO malignancy grades and histopathological subtypes. Expression of lncRNAs *MEG3*, *JPX*, *RNCR3*, and *ZFAS1* significantly differed between low and high malignancy grade (Mann–Whitney test; ∆∆C_T_ values) (Figure 3). *MEG3*, *JPX*, *RNCR3*, and *ZFAS1* showed significant decrease with tumour malignant progression.

**Figure 4:**
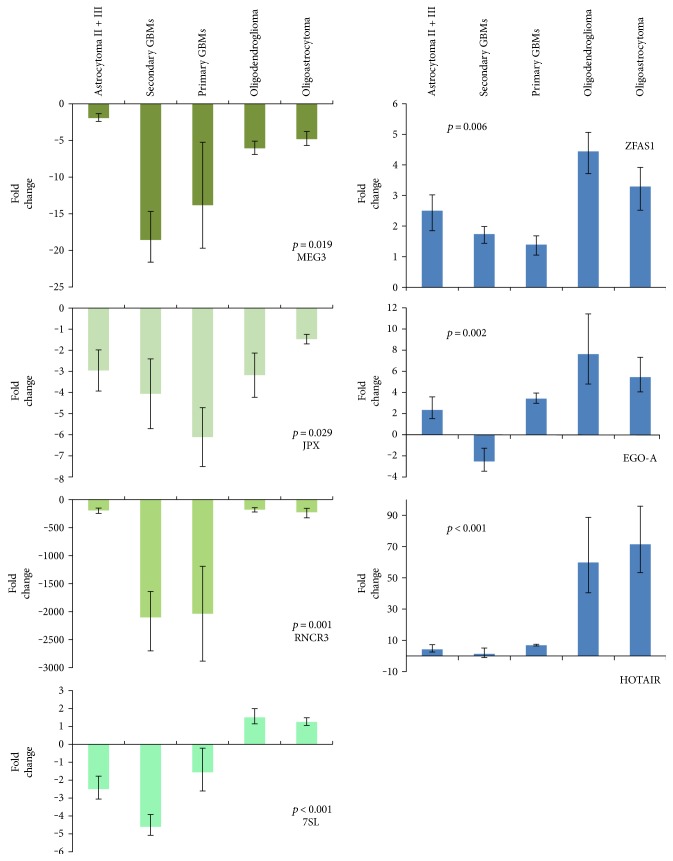
qPCR results of lncRNA gene expression in glioma subtypes. Expression analyses of glioma samples and human brain reference RNA for a subset of seven lncRNAs previously determined as differentially expressed. Data are presented as mean fold change (FC) ± standard FC error. *p* shows statistical significance for Kruskal-Wallis test of expression differences among all five subgroups (threshold set at *p* < 0.05).

Next, we grouped the samples upon their histological subtype into five groups and compared lncRNA's expression between all five using the Kruskal-Wallis test (∆∆C_T_ values). lncRNA expression patterns for corresponding glioma subtype are graphically presented in Figure 4. We observed significant differences across all samples for each of the lncRNAs. Expression of *HOTAIR* and *ZFAS1* was found overexpressed, and *MEG3*, *JPX*, and *RNCR3* were substantially underexpressed. *EGO-A* showed overexpression in all subtypes, except in secondary GBMs. Expression of *7SL* was found near normal level in oligodendroglioma and oligoastrocytoma, but underexpressed in astrocytoma, primary, and secondary GBMs.

### 3.3. There Are Distinctive lncRNA Expression Patterns between Glioma Subtypes

Kruskal-Wallis test does not tell between which subgroups the differences in expression significantly differ. Using the Mann–Whitney test, we compared the ∆∆C_T_ values of specific lncRNA of histologically determined subtypes among each other to establish subtype expression (Table 2) (for all graphic comparisons see graphs on Figure 4). *HOTAIR* exhibited much higher expression in oligodendroglioma and oligoastrocytoma compared to astrocytic tumours. *ZFAS1* was overexpressed in oligodendroglioma and oligoastrocytoma when cross tested with primary GBMs, which showed near normal expression levels. *MEG3* and *JPX* expression showed especially lower levels in primary GBMs compared to other astrocytic glioma. We also observed similar pattern for *RNCR3* with much lower expression levels in GBMs. And as observed before, expression of *MEG3*, *JPX*, and *RNCR3* decreases with malignancy grade (Figure 3). Significant differences were also found for *7SL* expression between oligodendroglioma and astrocytoma. Especially different pattern was observed for *EGO-A* in secondary GBMs.

### 3.4. Changed Expression of lncRNA-Related miRNA

In the subset of significantly changed lncRNAs we found that four are also encoding miRNAs. *7SL* was recently suggested to be regulated by *miR-125b* [29]. Wei et al. also suggest *miR-125b* to be a potential biomarker of glioma [30]. *HOTAIR* has been found to be coregulated with *miR-196a* [31], and genes for *RNCR3* and *MEG3* are acting as host genes for *miR-124a* and *miR-770* [32], respectively. We determined the expression levels of these four miRNAs and performed a lncRNA-miRNA linear correlation analysis to determine their possible relationship.

**Figure 5:**
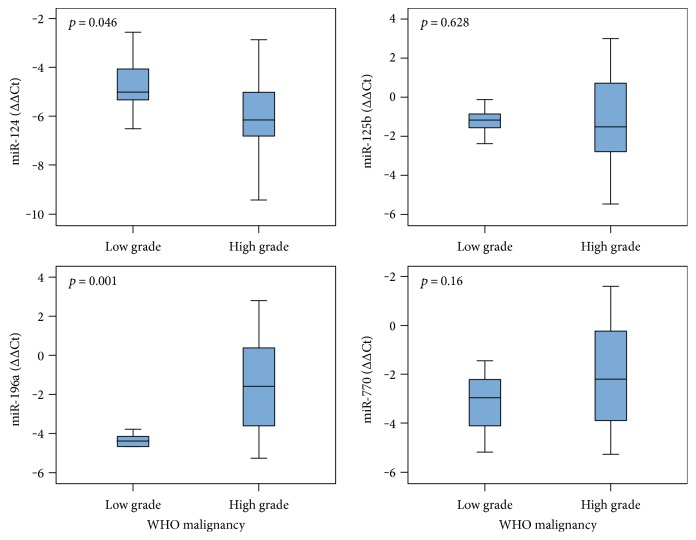
miRNA expression levels regarding the WHO malignancy grade. Expression analyses of four lncRNA-associated miRNAs between low and high malignancy grade glioma. Data are presented as ∆∆C_T_ box plots ± standard error.

**Figure 6:**
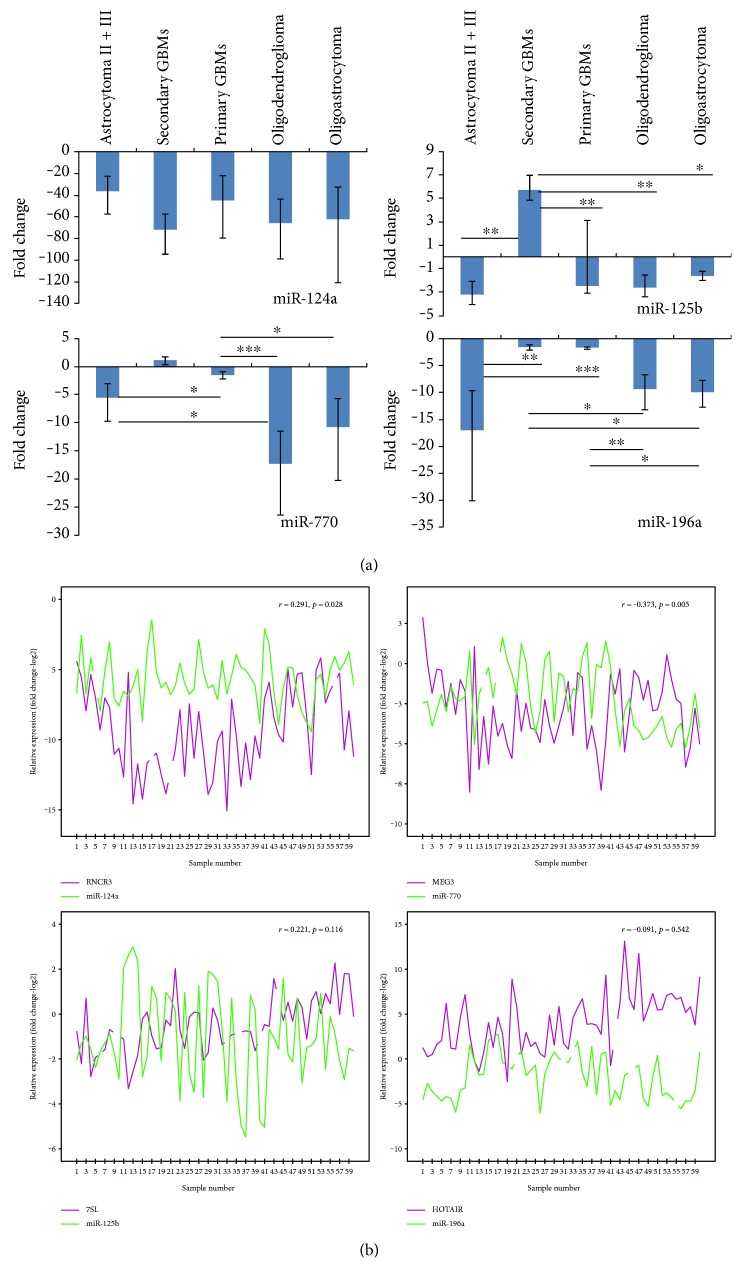
Expression results of miRNAs related to differentially expressed lncRNAs in glioma subtypes. (a) Expression analyses of miRNA, found to be associated with lncRNAs analysed, on the same cohort of sample as for lncRNAs as well as on human brain reference RNA. Data are presented as mean fold change (FC) ± standard FC error. Bars with asterisks represent significance for in-between-group comparison (^∗^0.01 ≤ *p* < 0.05; ^∗∗^0.001 ≤ *p* < 0.01; ^∗∗∗^*p* < 0.001). (b) Correlation relationship of expression levels in individual sample for miRNA and its related lncRNA. Letter *r* represents the magnitude of correlation and *p* the significance value.

Expression levels of *miR-124a* and *miR-196a* significantly differ between the WHO malignancy grades (Mann–Whitney test; ∆∆C_T_ values) (Figure 5). We observed significant differences in expression of *miR-770* among all glioma subtypes (*p* < 0.001). It has been decreased in all samples of astrocytoma, oligoastrocytoma, and oligodendroglioma and in majority of GBMs (expression varied). Expression significantly changed in oligodendroglioma and oligoastrocytoma compared to astrocytic subtypes (Figure 6(a)). *miR-196a* was also decreased in all groups (*p* = 0.001) and was significantly lower in oligodendroglioma, astrocytoma, and oligoastrocytoma compared to primary GBMs (2- to 4-fold) and secondary GBMs (up to 10-fold). In a proportion (~36%) of primary GBMs, *miR-196a* was slightly overexpressed (Figure 6(a)). For *miR-124a*, expression was decreased in all glioma subtypes when compared to reference RNA, but there was no significant change between different glioma subtypes. Also, *miR-125b* showed underexpression, except in secondary GBMs, for which we observed significant change comparing to other subtypes (*p* = 0.017) (Figure 6(a)). When comparing expression values of miRNAs between two different glioma subtypes, all miRNAs were shown to be differentially expressed between at least two glioma subtypes, except *miR-124a*, which did not reach statistically significant difference in expression between any two groups of samples (Figure 6(a)).

We observed moderate negative linear correlation (*r*_s_ = −0.373, *p* = 0.005) between expression of *miR-770* and *MEG3*, and positive correlation (*r*_s_ = 0.291, *p* = 0.028) between expression of *miR-124a* and *RNCR3* in all glioma samples. Both lncRNAs act as miRNA host genes. *miR-125b* and *7SL* and *miR-196a* and *HOTAIR* did not show significant correlation (Figure 6(b)). However, the lncRNA-miRNA relationship results provided are only suggestive as functional studies are required for stronger conclusion.

### 3.5. ncRNA's Expression Is Associated with Patients Age at Diagnosis and Survival Time

Expression of certain lncRNAs seems to be associated with patient's age at the time of diagnosis as correlation analysis showed moderate negative association for *MEG3* (*r*_s_ = −0.336, *p* = 0.009, *R*^2^ = 0.113), *ZFAS1* (*r*_s_ = −0.368, *p* = 0.004, *R*^2^ = 0.164), and *RNCR3* (*r*_s_ = −0.431, *p* = 0.001, *R*^2^ = 0.186).

Also, *miR-196a* showed moderate association with patient's age at time of diagnosis (*r*_s_ = 0.290, *p* = 0.039).

**Figure 7:**
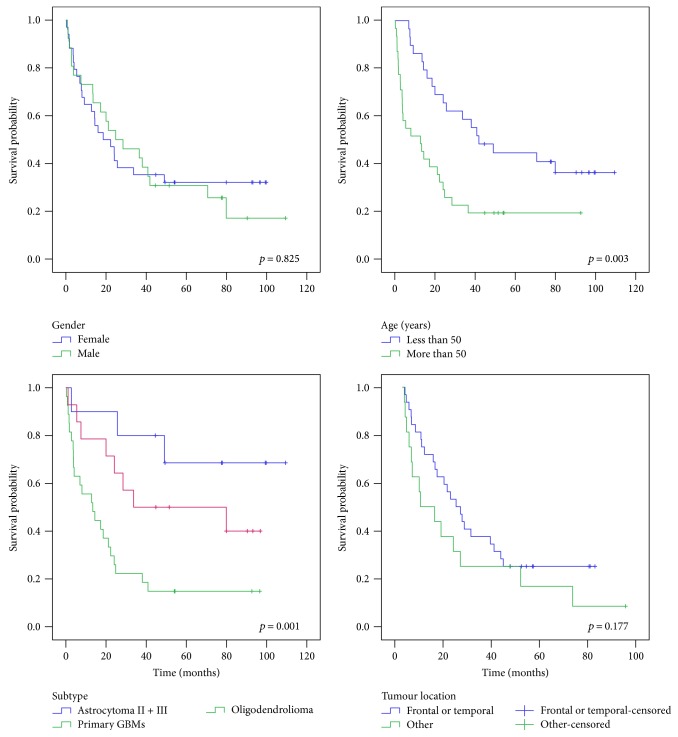
Kaplan-Meier survival curve analysis shows poor prognosis for patients older than 50 years at time of diagnosis and with diagnosed GBM.

**Figure 8:**
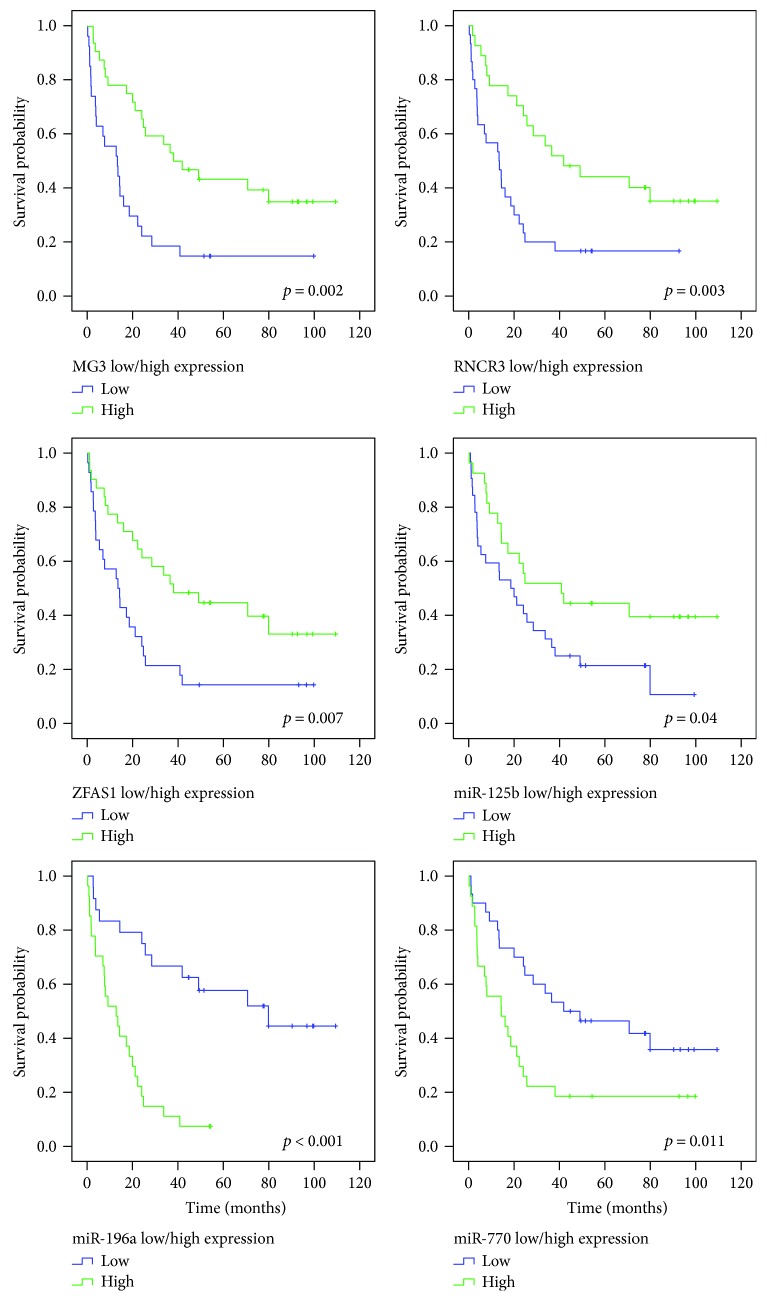
Expression of lncRNAs and miRNAs shows prognostic significance. Expression of individual sample was classified either low or high regarding the gene's ΔΔCt mean expression value.

The Kaplan-Meier estimate showed better survival for patients who were younger than 50 years at the time of diagnosis (Pearson's correlation *r*_s_ = −0.482, *p* < 0.001), but no difference regarding the sex or tumour location (Figure 7). Also, survival significantly differs between astrocytoma, primary GBMs, and oligodendroglioma. Because of a low sample number of oligoastrocytoma and secondary GBMs, we did not include them in the survival plot regarding the subtype (*p* = 0.001) (Figure 7). For determining possible association of gene's differential expression and survival, expression values of ncRNAs analysed were classified as low or high based upon individual ΔΔCt mean expression value. Kaplan-Meier plots and further analysis of survival differences (log-rank analysis) showed significant differences in survival for expression levels of *MEG3*, *ZFAS1*, *RNCR3*, *miR-770*, *miR-125b*, and *miR-196a* (Figure 8). These ncRNAs also showed significant association with survival when considering only the expression of astrocytoma, primary GBMs, and oligodendroglioma (data not shown). To identify the potential prognostic value of ncRNA expression, we used the univariate Cox proportional hazards regression analysis. We found significant association of survival with patient's age at diagnosis and expression of *MEG3*, *ZFAS1*, *RNCR3*, *miR-125b*, *miR-196a*, and *miR-770*, respectively. For the multivariate analysis, we took into consideration only the parameters with *p* < 0.05 in the univariate analysis. Results showed patient's age at diagnosis and expression of *ZFAS1*, *RNCR3*, *miR-125b*, and *miR-196a* as potential independent prognostic variables.

## 4. Discussion

Although many of lncRNAs known are involved in a variety of cancers, only a few have been associated with a particular cancer type [12]. lncRNA expression profiling revealed numerous lncRNA to be widely expressed in glioma, thus once again acknowledging the complexity of the brain and brain tumours and interweaving of different molecular levels. Tumour sample clustering based on expressional profile of 74 lncRNAs did not coincide with histopathological subtype, which suggests heterogeneous global expression of lncRNAs. However, determining glioma lncRNA expression profiles may be an especially helpful step in studying glioma expression network regarding lncRNAs' crucial regulatory roles [33]. And furthermore, expression patterns of specific lncRNAs or smaller gene-sets could potentially help us refine glioma subclassification, especially since one of the biggest problems in diagnosis is the morphological similarity of tumours. Indeed, Zhang et al. found novel tumour-related lncRNAs to be associated with malignancy grade and histological differentiation [13], and also our results indicate the existence of measurable differences of individual lncRNA or miRNA expression in glioma, also between histopathological subtypes and malignancy grades. Last but not the least, numerous lncRNAs show prognostic value in different human cancer types, including glioma [34–38]. Just recently, but in contrast to our findings, Gao et al. found elevated expression of *ZFAS1* in glioma as an independent, unfavourable prognostic factor [39]. Similarly, increased expression of *NEAT1* [40], *HOTAIR* [41], and *MALAT1* [23, 38] was also found associated with poor prognosis in glioma. Zhang et al. explored a six-lncRNA signature as a set of prognostic genes in glioma—*PART1*, *MGC21881*, *MIAT*, *GAS5*, and *PAR5* were correlated with prolonged survival and *KIAA0495* in association with the poorer [34].

Among seven validated lncRNAs, there is already a considerable research and data for roles of *HOTAIR* and *MEG3* in various cancer types [31, 42–52]. And just recently, Gao et al. reported *ZFAS1*'s oncogenic potential in glioma tumours [39]. lncRNAs can exhibit oncogenic or tumour suppressive functions [11]. Regarding processes of tumour growth, all three were found to be involved in cell proliferation in glioma [39, 53–55] and various other cancer types [56–61]. As an oncogenic lncRNA, *HOTAIR* is involved in epigenetic gene silencing, cell growth, and progression of various cancers [42–44]. A number of studies show that increased expression of *HOTAIR* enhances cell proliferation and invasion of cancer cells and has been associated with tumour progression and prognosis [45, 55, 56, 59, 62]. Overall high expression of *HOTAIR* in our glioma samples indicates its association also with glioma development and was already found essential for glioblastoma proliferation [55, 62, 63]. Expression of *HOTAIR* has been found to differentiate between astrocytoma, oligodendroglioma, and/or oligoastrocytoma, suggesting its use as a potential biomarker in distinguishing morphologically similar cases [41].

On the contrary, *MEG3* expression has been strongly decreased in glioma and its expression decreases with malignancy grade, thus acting as a tumour suppressor and contributing to glioma progression [18, 64]. As a putative tumour suppressor, it inhibits cell proliferation/DNA synthesis by stimulating the expression of tumour suppressor p53 [65] and modulating the binding of p53 on the promoter of its target genes [66]. On the other hand, *MEG3* is also able to inhibit cell proliferation and promote cell apoptosis in the p53-independent manner [64]. In line with the latter, its decreased levels are also associated with poorer survival. Overexpression of *MEG3* in human glioma cell lines inhibits cell proliferation and promotes cell apoptosis [18, 64].

Also found to be involved in p53-dependent cell cycle control is *ZFAS1* [54], an antisense lncRNA localized at the 5′ end of the protein-coding gene *ZNFX1* [67] that is widely expressed in numerous tissues, including the brain (http://www.proteinatlas.org). In their research of *ZFAS1* in glioma tissues and cell lines, Lv et al. [68] and Gao et al. [39] found its increased expression correlates with tumour stage and poor survival, matching with our results. Significantly increased *ZFAS1* expression indicates its oncogenic function [54]—its silencing decreases cell's proliferation through G1 cell cycle arrest [68]. Moreover, by regulating the epithelial-mesenchymal transition (EMT) and Notch signalling pathway, it could contribute to glioma progression [39]. In addition to lncRNA's oncogenic or tumour suppressive effect, one can also exhibit both effects [3, 5, 11]. *ZFAS1* was found downregulated in breast carcinoma, which suggests *ZFAS1* also as a possible tumour suppressor [67].

On the contrary to abovementioned lncRNAs, only little or none is known about the expression and function of lncRNAs *EGO-A*, *RNCR3*, *JPX*, and *7SL* in glioma. Abdelmohsen et al. reported *7SL* to be highly expressed in various cancer tissues and by repressing *p53* mRNA translation consequently promoting cancer cell growth [69]. *7SL* is a small RNA component of cytoplasmic SRP (signal recognition particle) complex, a guide that directs nascent secretory proteins towards the endoplasmic reticulum and is necessary for synthesis of normal, active proteins. It is essential for translocation across the membrane of endoplasmic reticulum [70], and its dysregulation may possibly result in impaired protein synthesis machinery. Thus, it is not surprising that *7SL* is ubiquitously expressed in numerous tissues, including the brain [71]. Our study showed decreased *7SL* levels in astrocytic tumours and increased levels in oligodendroglioma and oligoastrocytoma. These expression differences between oligodendroglioma and astrocytoma may be an additional genetic parameter for distinguishing these subtypes among each other, especially since expression is near normal levels in oligodendroglioma (and oligoastrocytoma).

Expression levels of *EGO-A* (eosinophil granule ontogeny isoform A) were significantly increased in all glioma subtypes, with the exception of secondary GBMs, which can be probably attributed to the small sample number. It is worth mentioning that secondary GBMs are relatively rare, partly because precursor low-grade or anaplastic astrocytoma develop at younger age than other GBMs, and some patients succumb before the disease progresses, and partly because they are mistakenly classified as primary GBMs [72]. *EGO-A* function is not yet known [73], but its potential role in glioma biogenesis may be implied by the chromosomal location, since its host gene *ITPR1* (inositol triphosphate receptor type 1) is encoded in close proximity to *EGR-1* (early growth response 1), a transcriptional regulator of genes required for induction of mitosis, cell differentiation, and growth [74]. Expression of *EGR-1* gene in glioma cells is induced by overexpression of *EGFR* and *PDGFR* genes, thus suggesting *EGR-1* as a connection of growth factor stimulation with gene expression changes [74]. Association of *EGR-1* gene with expression of growth factor might also explain the differences in expression level of *EGO-A* between the two GBM subtypes (small sample number aside), since *EGFR* overexpression is typical for primary GBMs, but is rare in secondary GBMs [72]. However, whether the correlation between expression levels of *EGR-1* and *EGO-A* loci exists is yet to be determined. Xu et al. found expression of *EGO* transcript to be downregulated in breast cancer and to play an important role in progression of breast cancer and prognosis, thus serving as potential prognostic target [75].

*RNCR3* (retinal noncoding RNA 3) is highly expressed in the brain, yet we have not found any reported studies of its expression in glioma, and to date very little is known about its biological function. Especially, it was identified as a precursor of the *miR-124a*, the most abundant miRNA in the vertebrate CNS and necessary for normal brain development [76]. Expression level of *miR-124a* was significantly decreased in anaplastic astrocytoma and GBMs, when compared to normal brain tissue [77], and also in oligodendroglioma [78]. We found substantially decreased expression of *miR-124a* and *RNCR3* in primary and secondary GBMs and a positive correlation between expression of these two ncRNAs. Since the *RNCR3* is a precursor for *miR-124a*, decreased expression of *miR-124a* could be a possible consequence of reduced *RNCR3* expression. Low expression of *RNCR3* also indicates lower survival probability. Regarding the findings, we can infer that *RNCR3* and its encoded *miR-124a* may be implicated in the development and progression of glioma. *miR-124a* is the most extensively investigated miRNAs in glioma cell proliferation. In the earliest study, it has been shown that upregulation of miR-124a induces glioblastoma cell cycle arrest. These results suggested that targeted delivery of *miR-124* to glioblastoma tumour cells may be therapeutically efficacious for the treatment of this disease [77]. Fowler et al. [79] found an ectopic expression of *miR-124a* significantly inhibiting GBM migration and invasion, which once more supports its role in glioma progression. Moreover, restoration of *miR-124a* could inhibit glioma cell proliferation and invasion through *miR-124a* blocking the expression of the *IQGAP1* gene and downstream *β*-catenin and cyclin D1 [80] and *PIM1* in astrocytoma cancer cells [81]. Additionally, transfection with *miR-124* inhibitor rescued the proliferative ability of human glioma cells. Results demonstrated that miR-124 is an important downstream target gene of Hedgehog signalling and that *glioma-associated oncogene-miR-124-AURKA* axis is essential for the proliferation and growth of human glioma cells [82].

Expression of miR-770 showed decreased levels in lower malignancy grades and a better survival probability. The expression was in an inverse correlation to its host gene, that is, MEG3, and regarding the findings of miR-770 being a downstream transcriptional target of Wnt/*β*-signalling pathway [83], it is reasonable to propose for MEG3 and miR-770 possibly having different mechanisms regulating their expression. There are only few investigations performed about the role of *miR-770* in carcinogenesis; moreover, only one study is investigating its role in proliferation. By acting as a sponge for *miR-770*, leading to its downregulation, lncRNA *PCGEM1* stimulates proliferation of osteoarthritic synoviocytes [84]. Whether or not this is also the case in glioma cells is yet to be investigated. Analyses of miRNA-lncRNA-mRNA interactions using their expression profiles alone are not enough to further understand the potential relationships between different RNA molecules [85]. Differences in lncRNA and its associated miRNAs' expression can also be the consequence of different stability of the transcript, splicing, promoter methylation, or miRNA's maturation [26].

*miR-196a* is concurrently upregulated with *HOTAIR* in gastrointestinal stromal tumours (GISTs) [31], but was downregulated in our glioma subtypes, with no association to *HOTAIR*. It has been shown that *miR-196a* is expressed in lower levels in oligodendrogliomas, astrocytomas, and oligoastrocytomas compared to glioblastoma, similar to our results [86]. *miR-196a* expression seems to be highly associated with patient's survival as its higher expression indicates poorer prognosis and shorter survival time, coinciding with HOTAIR overexpression survival prediction and their suggested coregulated expression [41]. Investigating the oncogenic effects of *miR-196a in vivo* and *in vitro* revealed that it induces and promotes proliferation and suppresses apoptosis through inhibition of the I*κ*B*α* [87].

We were not able to detect any significant correlation between expression patterns of *miR-125b* and *7SL*, although there is an inverse overall expression in oligoastrocytoma, oligodendroglioma, and secondary GBMs. *miR-125b* has already been shown to be upregulated in primary GBMs (but downregulated in GBM cell lines) [88] and in oligodendroglioma [78]. Its overexpression promotes glioma cell proliferation and inhibits cell apoptosis, thus supporting the suggested oncogenic role [89, 90].

## 5. Conclusions

Studies of lncRNAs and miRNAs over the past decade increasingly reveal the importance of ncRNAs in cancer development and progression. All in all, our results corroborate the involvement of noncoding RNAs in the complex nature of primary brain tumours. We show that several lncRNAs, some already related to glioma and some investigated for the first time, could importantly contribute to glioma development. Expression level of several lncRNAs and certain lncRNA-associated miRNAs significantly changes between the analysed glioma subtypes and malignancy grades. Moreover, their possible correlation with miRNAs suggests a complex interplay of lncRNA-miRNA in regulating gene expression in glioma. Observing high expressional variability of the results opens numerous further questions and presents the foundation for further research of noncoding RNA implication in glioma formation and development, which will conceivably provide new glioma-specific biomarkers and targets for treatment of the disease. Although our research does not show/determine the exact mechanism of ncRNAs in tumour development, it does show that the analyses of expressional screening represent guidelines for further research and are efficient in the search of new cancer-related genes. It has been already accepted that lncRNAs have an immense role in glioma pathophysiology, but the question is which lncRNAs are implicated in malignant transformation and how they contribute to each level of tumorigenesis.

## Supplementary Material

Table S1. Calculated log2(Fold change) values, obtained by lncRNA profiler using the qPCR method, on the primary set of glioma samples of different, histology-defined subtypes.

## Figures and Tables

**Table 1:** Clinicopathological characteristics of patients in the study.

Patients' demographics		
Number of patients	64	
Sex (female/male)	36/28 (1.3 : 1)	
Median age at diagnosis (years) (min.–max.)	51.8 (3.9–83.1 years)	
Number < 50 years	29	
Number > 50 years	35	
Glioma classification	*Glioma subtype*	*WHO grade*
(i) Astrocytoma (AC)	4 diffuse AC	WHO II
	6 anaplastic AC	WHO III
	4 secondary GBM	WHO IV
	31 primary GBM	
(ii) Oligodendroglioma (ODG)	14 anaplastic ODG	WHO III
(iii) Oligoastrocytoma (OAC)	5 anaplastic OAC	WHO III
Tumour location		
(i) Frontal or temporal	32	
(ii) Other regions	16 (16 na)	

na: not applicable.

**Table 2:** Significantly different expression of lncRNA between glioma subtypes (represented as *p* value).

Mann–Whitney 2-independent test	Secondary glioblastoma	Primary glioblastoma	Anaplastic oligodendroglioma	Anaplastic oligoastrocytoma
Grade II + III astrocytoma	*EGO-A* 0.016	*RNCR3* 0.001 *MEG3* 0.001	*HOTAIR* 0.004 *EGO-A* 0.010 *7SL* 0.005	*EGO-A* 0.024 *7SL* 0.038
	Secondary glioblastoma	*HOTAIR* 0.039 *EGO-A* 0.006 *7SL* 0.011	*HOTAIR* 0.003 *EGO-A* 0.004 *7SL* 0.003	*HOTAIR* 0.034 *EGO-A* 0.021 *7SL* 0.021
		Primary glioblastoma	*HOTAIR* 0.001 *RNCR3* 0.001 *ZFAS1* 0.001 *JPX* 0.019 *7SL* < 0.001	*HOTAIR* 0.041 *RNCR3* 0.018 *ZFAS1* 0.036 *JPX* 0.014
			Anaplastic oligodendroglioma	/
